# Estradiol and Hyperhomocysteinemia Are Linked Predominantly Through Part Renal Function Indicators

**DOI:** 10.3389/fendo.2022.817579

**Published:** 2022-05-18

**Authors:** Xiao Na Niu, He Wen, Nan Sun, Yi Yang, Shi Hong Du, Rong Xie, Yan Nan Zhang, Yan Li, Xiu Qin Hong

**Affiliations:** ^1^ First Affiliated Hospital of Hunan Normal University, Hunan Provincial People’s Hospital, Changsha, China; ^2^ Department of Cardiology, The Second Affiliated Hospital of Air Force Military Medical University, Tangdu Hospital, Xi An, China

**Keywords:** estradiol (E2), hyperhomocysteinemia (HHcy), homocysteine (Hcy), mediation effect, case–control study

## Abstract

**Background:**

Previous studies have shown that estrogen, kidney function, and homocysteine (Hcy) or hyperhomocysteinemia (HHcy) are related to each other. However, the underlying biological mechanisms still remain unclear. We aimed to explore the association between estradiol (E2) and HHcy in the female population, and to further evaluate the mediating role of renal function indicators.

**Methods:**

This unmatched case–control study consisted of 1,044 female participants who were 60.60 ± 12.46 years old. Data on general demographic characteristics, such as age, smoking and drinking status, menopause and so on were collected in a personal interview, and laboratory examinations were performed by well-trained personnel. The mediating effect model was applied to analyze the direct and indirect effects of E2 on Hcy.

**Results:**

The average levels of Hcy and E2 of the participants were 12.6 μmol/L and 14.95 pg/ml. There were statistical differences in renal indexes blood urea nitrogen (BUN), serum creatinine (Scr), uric acid (UA), glomerular filtration rate (GFR) and E2 between HHcy group and non-HHcy group. The logistic regression models showed that UA was risk factor for HHcy (P <0.001), GFR and E2 were protective factors for HHcy after adjusting for confounding factors (P <0.001). The indirect effects of E2 on Hcy through UA and GFR accounted for 14.63 and 18.29% of the total impacts of E2 on Hcy.

**Conclusions:**

These data indicated that E2 was a protective factor of HHcy, and the effects of E2 on HHcy may be mediated by renal function indicators UA and GFR.

## Introduction

The molecular formula of homocysteine (Hcy) is C4H9NO2S, which is an intermediate product of cysteine metabolism and methionine cycle (1, 2). There are three forms of Hcy in the body. Approximately 70–80% of Hcy is in the form of plasma protein; 20–30% of Hcy exists in the form of dimer. The third is free form, accounting for 1–2% (3). In normal adults, the fasting plasma Hcy concentration is maintained between 5 and 15 μmol/L. When the plasma Hcy concentration in the body exceeds the normal physiological concentration range, it is called HHcy (4). Chinese hypertensive patients have high Hcy and about 75% of hypertensive patients have elevated Hcy (5, 6). Elevated Hcy seem to be more common in China than anywhere in the world. This may be closely related to the genetic characteristics, lifestyle and folic acid deficiency of the Chinese population (7, 8). Many studies have confirmed that Hcy was closely related to the occurrence and development of cardiovascular and cerebrovascular diseases, diabetes, chronic kidney diseases, neurological diseases and other diseases, and experimental studies have already indicated that Hcy can cause vascular endothelial damage, leading to vascular damage (9–12).

Estrogen is a key type of fat-soluble steroid hormone, mostly produced by the ovary, testis and adrenal cortex, mainly estradiol (E2), estriol, and estrone. E2 is the richest and most active one, which has the physiological effects of promoting the maturation of female organs, maintaining sexual and reproductive functions, and also has the functions of regulating lipid metabolism, protecting the cardiovascular system, and resisting platelets. Some investigators performed oral methionine load testing in 46 premenopausal (27–44 years) and 26 postmenopausal (50–60 years) women and measured plasma Hcy and estrogen levels after taking methionine on an empty stomach for 6 h. Finally, it was found that the levels of estrogen of postmenopausal women were significantly reduced, and Hcy was generally increased. The above results suggested that estrogen may affect the metabolism of Hcy, the above process can be partially reversed by supplementing estrogen (13, 14). A study has shown that exogenous estrogen administered in a clinically relevant manner can act as an effective kidney protector after systemic ischemia–reperfusion (15). In the experiment of animal kidney injury, female mice supplemented with E2 recovered better than older female mice, which proved E2 has a positive effect on kidney function (6). In addition, there are many enzymes related to Hcy metabolism in kidney tissue. When renal function is impaired, Hcy metabolism is abnormal (16). However, few studies have analyzed the relationship between the E2, kidney indicators, and Hcy. This study decided to analyze the relationship between the three through the mediating effect model.

## Materials and Methods

### Study Design and Population

Our study used a non-matched case-control study with 316 HHcy female patients in the case group and 728 female patients in the control group respectively in the Hunan Provincial People’s Hospital from July 2018 to November 2019. The female patients of HHcy in the case group were diagnosed by licensed medical practitioners. At the same time, this study excluded pregnant and lactating women; patients with chronic systemic diseases such as severe heart, kidney, blood system, and patients with malignant tumor, severe infection, anemia and other diseases; patients who have recently used sex hormone, fibrinolytic and anticoagulant drugs, and drugs that may affect blood lipids and Hcy. This study was approved by the Institutional Ethics Review Board of Hunan Provincial People’s Hospital, Changsha, China. All subjects in this study signed the written informed consent.

### Data Collection

Baseline characteristics of participants were obtained by well-trained experimental personnel using a standard questionnaire in a personal interview. Before the survey was performed, all eligible investigators were invited to attend the standard training. All participants were asked to fast for at least 12 h before taking a venous blood sample. The professionally trained nurses used disposable blood draw needles and strictly followed the standard of sterile blood collection, collecting fasting venous blood in the morning. Lipid indexes (TG Triglyceride, TC Total cholesterol, LDL-C Low density lipoprotein cholesterol, HDL-C High density lipoprotein cholesterol), liver function indexes (ALT Alanine aminotransferase, AST Aspartate aminotransferase, ALB Albumin, GLB Globulin), kidney function indexes (BUN, Scr, UA), E2 and testosterone (T) were measured by standard laboratory procedures. GFR was calculated by a recognized formula. Those who drank regularly for more than 6 months were assigned to the drinking group. Current smokers (at least one per day and consecutive cigarettes for one year) and former smokers were divided into smokers group. When enrolled female patients have continuous amenorrhea for 12 months, this was defined as menopause. BUN, Scr, UA and GFR grouping were based on the reference range of the Hunan Provincial People’s Hospital. Because the BUN levels of the study subjects were greater than 1.70 mmol/L, our study divided the BUN levels of female patients into two groups (1.70–8.30 mmol/L; >8.30 mmol/L).

### Statistical Analysis

Qualitative variables were expressed as number and percentage. Normally distributed quantitative variables were presented as mean ± standard deviation (SD). Two-sample t-tests for normally distributed quantitative variables and the χ^2^ test for qualitative variables were used to compare differences between the two groups. Use median and interquartile range (IQR) to describe the centralized and discrete trends of quantitative variables that were not normally distributed. The non-parametric test was used to compare the differences in quantitative variables that were not normally distributed between the two groups. Logistic regressions were used to assess the relationship between renal function indicators, E2 and HHcy. In the mediating effect model, the relationship between the independent variable X, the intermediate variable M, and the dependent variable Y can be expressed by the following regression equation: (1): Y = cX + e_1_; (2): M = aX + e_2_; (3): Y = b M + c′;X + e_3_ (c = total effect, a = indirect effect 1, b = indirect effect 2, and c′; = direct effect, e_1_–e_3_ represent the regression residual, mediation effect = (ab/c) × 100%). Statistical analysis was performed with SPSS 23.0 software package (SPSS, Chicago, Illinois, USA) and a 2-tailed P <0.05 was considered to be statistically significant.

## Results

### Characteristics of Female Patients

**Figure 1:**
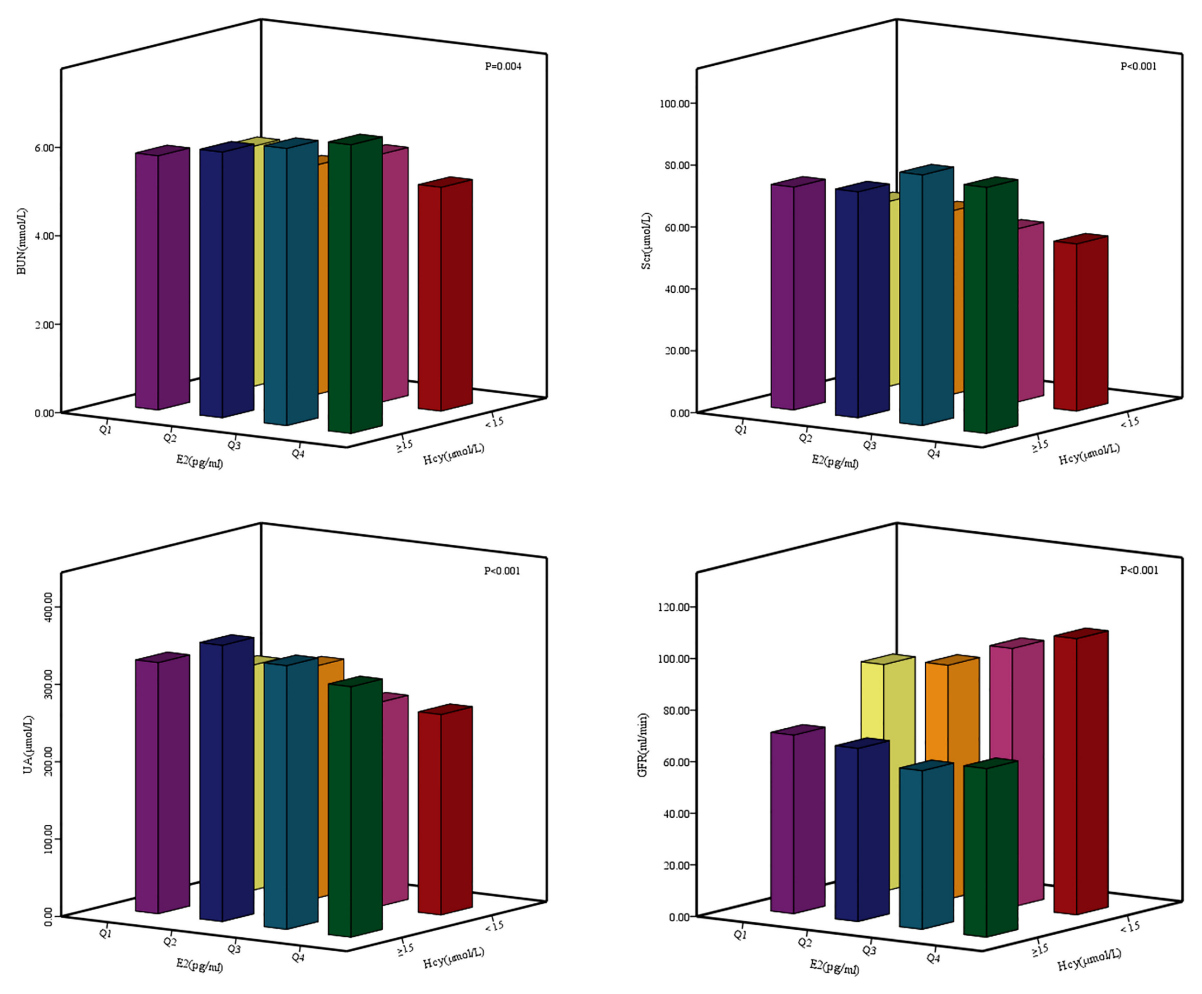
Differences of renal function indexes BUN, Ser, UA, GFR between different E2 and Hey levels groups. E2: Q1: ≤ 4.99 pg/ml; Q2: 4.99 pg/ml - 14.95 pg/ml; Q3: 14.95 pg/ml - 26.80 pg/ml; Q4: > 26.80 pg/ml.

The age of the female patients in this study was 60.60 ± 12.46 years old, the average age of patients in the case group was greater than that of the control group (P <0.001). The BMI of the two groups were 23.87 ± 7.50 kg/m^2^ and 23.73 ± 3.17 kg/m^2^ respectively. There was no statistical difference in BMI between the two groups (P = 0.755). In the total study population, 61.11% of women were urban area, 2.78% of study subjects were smokers; 3.83% of women were drinkers; 83.43% of women were menopausal female patients. Less than high school, high school and above high school groups were 604 (57.85%), 273 (26.15%) and 167 (16.00%). There were no statistically significant difference in TC and HDL-C between two groups (P_TC_ = 0.142; P_HDL-C_ = 0.215). The LDL-C of the case group was higher than that of the control group (P = 0.003). The TG of the case group was higher than that of the control group (P = 0.001). The T in the case group was higher than that in the control group (P <0.001) (**Table 1**). The median and interquartile ranges (IQR) of the BUN of the participants were 5.52 mmol/L and 4.37–7.50 mmol/L. The BUN of the case group was higher than that of the control group (P = 0.001). The Scr of the case group was higher than that of the control group (P <0.001). The median and IQR of the UA of the participants were 295.00 μmol/L and 226.25–361.15 μmol/L. The UA of the case group was higher than that of the control group (P <0.001). The median and IQR of the GFR of the participants were 91.00 ml/min and 69.43–105.72 ml/min. The GFR of the case group was lower than that of the control group (P <0.001). The E2 of the case group was lower than that of the control group (P <0.001) (**Table 2**). There were differences in the BUN, Scr, UA, GFR between the different E2 and the Hcy levels group (**Figure 1**).

**Table 1:** Differences in general demographic data and clinical indicators of the study population.

Characteristics	All participants (N = 1044)	Hcy <15 μmol/L (N = 728)	Hcy ≥15 μmol/L (N = 316)	*P-*value	OR (95% CI)
Living area				0.028	
Urban area	638 (61.11%)	429 (58.93%)	209 (66.14%)		1
Rural area	406 (38.89%)	299 (41.07%)	107 (33.86%)		0.735 (0.557, 0.968)
Smoking history				0.750	
Yes	29 (2.78%)	21 (2.88%)	8 (2.53%)		1
No	1,015 (97.22%)	707 (97.12%)	308 (97.47%)		1.144 (0.501, 2.610)
Drinking history				0.754	
Yes	40 (3.83%)	27 (3.71%)	13 (4.11%)		1
No	1,004 (96.17%)	701 (96.29%)	303 (95.89%)		0.898 (0.457, 1.764)
Menopause				<0.001	
Yes	871 (83.43%)	569 (78.16%)	302 (95.57%)		1
No	173 (16.57%)	159 (21.84%)	14 (4.43%)		0.166 (0.094, 0.292)
Education				0.058	
Less than high school	604 (57.85%)	404 (55.49%)	200 (63.29%)		1
High school	273 (26.15%)	199 (27.34%)	74 (23.42%)		0.751 (0.548, 1.030)
Above high school	167 (16.00%)	125 (17.17%)	42 (13.29%)		0.679 (0.460, 1.001)
Age (years)	60.60 ± 12.46	57.59 ± 11.91	67.53 ± 10.86	<0.001	
≤49	172 (16.47%)	156 (21.43%)	16 (5.06%)	<0.001	1
50–59	319 (30.56%)	265 (36.40%)	54 (17.09%)		1.987 (1.099, 3.591)
≥60	553 (52.97%)	307 (42.17%)	246 (77.85%)		7.813 (4.548, 13.422)
BMI (kg/m^2^)	23.83 ± 6.50	23.87 ± 7.50	23.73 ± 3.17	0.755	
<24.0	605 (57.95%)	429 (58.93%)	176 (55.70%)	0.331	1
≥24.0	439 (42.05 %)	299 (41.07%)	140 (44.30%)		1.141 (0.874, 1.490)
TG (mmol/L)	1.47 (1.04, 2.08)	1.40 (1.00, 1.99)	1.66 (1.14, 2.29)	0.001	
Quartile 1	268 (25.67%)	203 (27.89%)	65 (20.57%)	0.003	1
Quartile 2	255 (24.43%)	188 (25.82%)	67 (21.20%)		1.113 (0.750, 1.652)
Quartile 3	263 (25.19%)	175 (24.04%)	88 (27.85%)		1.570 (1.075, 2.294)
Quartile 4	258 (24.71%)	162 (22.25%)	96 (30.38%)		1.851 (1.270, 2.697)
TC (mmol/L)	4.52 ± 1.12	4.49 ± 1.13	4.60 ± 1.08	0.142	
Quartile 1	264 (25.29%)	198 (27.20%)	66 (20.89%)	0.041	1
Quartile 2	259 (24.81%)	176 (24.18%)	83 (26.26%)		1.415 (0.966, 2.072)
Quartile 3	262 (25.09%)	188 (25.82%)	74 (23.42%)		1.181 (0.802, 1.739)
Quartile 4	259 (24.81%)	166 (22.80%)	93 (29.43%)		1.681 (1.153, 2.450)
LDL-C (mmol/L)	2.70 ± 0.88	2.65 ± 0.88	2.82 ± 0.87	0.003	
Quartile 1	261 (25.00%)	195 (26.79%)	66 (20.89%)	0.028	1
Quartile 2	262 (25.09%)	187 (25.69%)	75 (23.74%)		1.185 (0.805, 1.745)
Quartile 3	265 (25.39%)	185 (25.41%)	80 (25.31%)		1.278 (0.871, 1.874)
Quartile 4	256 (24.52%)	161 (22.11%)	95 (30.06%)		1.743 (1.196, 2.542)
HDL-C (mmol/L)	1.25 ± 0.32	1.26 ± 0.32	1.23 ± 0.33	0.215	
Quartile 1	271 (25.96%)	182 (25.00%)	89 (28.16%)	0.368	1
Quartile 2	255 (24.43%)	172 (23.62%)	83 (26.26%)		0.987 (0.685, 1.421)
Quartile 3	267 (25.57%)	195 (26.79%)	72 (22.79%)		0.755 (0.521, 1.094)
Quartile 4	251 (24.04%)	179 (24.59%)	72 (22.79%)		0.823 (0.566,1.194)
ALT (U/L)	16.00 (11.73, 24.00)	16.60 (12.00, 24.10)	15.00 (11.00, 23.40)	0.037	
Quartile 1	261 (25.00%)	172 (23.62%)	89 (28.16%)	0.005	1
Quartile 2	263 (25.19%)	168 (23.08%)	95 (30.06%)		1.093 (0.763, 1.565)
Quartile 3	268 (25.67%)	205 (28.16%)	63 (19.94%)		0.594 (0.406, 0.869)
Quartile 4	252 (24.14%)	183 (25.14%)	69 (21.84%)		0.729 (0.500, 1.062)
AST (U/L)	20.00 (16.63, 25.00)	19.85 (16.50, 24.97)	20.00 (17.00, 25.52)	0.814	
Quartile 1	261 (25.00%)	189 (25.96%)	72 (22.79%)	0.414	1
Quartile 2	284 (27.20%)	189 (25.96%)	95 (30.06%)		1.319 (0.914, 1.904)
Quartile 3	244 (23.37%)	175 (24.04%)	69 (21.84%)		1.035 (0.701, 1.527)
Quartile 4	255 (24.43%)	175 (24.04%)	80 (25.31%)		1.200 (0.821, 1.753)
ALB (g/L)	40.27 ± 4.48	40.22 ± 4.53	40.36 ± 4.37	0.640	
Quartile 1	264 (25.29%)	182 (25.00%)	82 (25.95%)	0.009	1
Quartile 2	261 (25.00%)	203 (27.89%)	58 (18.35%)		0.634 (0.429, 0.938)
Quartile 3	262 (25.09%)	171 (23.49%)	91 (28.80%)		1.181 (0.821, 1.700)
Quartile 4	257 (24.62%)	172 (23.62%)	85 (26.90%)		1.097 (0.759, 1.585)
GLB (g/L)	25.07 ± 4.43	24.48 ± 4.31	26.43 ± 4.39	<0.001	
Quartile 1	261 (25.00%)	211 (28.98%)	50 (15.82%)	<0.001	1
Quartile 2	271 (25.96%)	205 (28.16%)	66 (20.89%)		1.359 (0.897, 2.057)
Quartile 3	254 (24.33%)	167 (22.94%)	87 (27.53%)		2.198 (1.470, 3.289)
Quartile 4	258 (24.71%)	145 (19.92%)	113 (35.76%)		3.289 (2.217, 4.879)
T (ng/ml)	0.41 (0.31, 0.57)	0.39 (0.30, 0.50)	0.49 (0.38, 0.68)	<0.001	
Quartile 1	262 (25.09%)	209 (28.71%)	53 (16.77%)	<0.001	1
Quartile 2	269 (25.77%)	213 (29.26%)	56 (17.72%)		1.037 (0.680, 1.580)
Quartile 3	261 (25.00%)	171 (23.49%)	90 (28.48%)		2.075 (1.398, 3.081)
Quartile 4	252 (24.14%)	135 (18.54%)	117 (37.03%)		3.418 (2.314, 5.048)

BMI, Body Mass Index; TG, Triglyceride; TC, Total cholesterol; LDL-C, Low density lipoprotein cholesterol; HDL-C, High density lipoprotein cholesterol; ALT, Alanine aminotransferase; AST, Aspartate aminotransferase; ALB, Albumin; GLB, Globulin; T, Testosterone.

**Table 2:** Differences of renal function indicators and estradiol in the study population.

Variables	Normal range	All participants		Hcy <15 μmol/L		Hcy ≥15 μmol/L		*P* value
		Median	IQR	Median	IQR	Median	IQR	
BUN (mmol/L)	1.70–8.30	5.52	4.37–7.50	5.30	4.17–7.32	5.97	4.74–7.79	0.001
Scr (μmol/L)	40–100	60.00	50.83–73.00	56.70	49.00–66.28	73.50	60.00–93.00	<0.001
UA (μmol/L)	155–357	295.00	226.25–361.15	274.60	202.55–336.00	337.00	273.25–410.75	<0.001
GFR (ml/min)	90–110	91.00	69.43–105.72	98.53	83.40–108.40	67.95	51.58–86.75	<0.001
E_2_ (pg/ml)	–	14.95	4.99–26.80	19.00	8.52–29.40	8.60	4.99–16.62	<0.001

BUN, Blood urea nitrogen; Scr, Serum creatinine; UA, Uric acid; GFR, Glomerular filtration rate; E_2_, Estradiol.

### The Association Between Renal Function Indexes, E2 and HHcy

UA was correlated with HHcy, UA was risk factors for HHcy. The risk of HHcy of in the UA range of 155–357 μmol/L group was 3.320 times that of the <155 μmol/L group after adjusting for covariates (95% CI: 1.878–5.869; P <0.001). The risk of HHcy of UA >357 μmol/L group was 8.602 times that of UA <155 μmol/L group after adjusting for covariates (95% CI: 4.760–15.547; P <0.001). GFR and E2 were correlated with HHcy, GFR and E2 were protective factors for HHcy. The risk of HHcy in the GFR range of 90–110 ml/min group was 83.3% lower than that of GFR <90 ml/min group after adjusting for covariates (95% CI: 0.118–0.238; P <0.001). The risk of HHcy of GFR >110 ml/min group was 79.6% lower than that of GFR <90 ml/min group after adjusting for covariates (95% CI: 0.119–0.350; P <0.001). The risk of HHcy in the E2 range of 4.99–14.95 pg/ml group was 0.534 times than that of E2 ≤4.99 pg/ml group after adjusting for covariates (95% CI: 0.374–0.762; P <0.001). The risk of HHcy in the E2 range of 14.95–26.80 pg/ml group was 0.230 times than that of E2 ≤4.99 pg/ml group after adjusting for covariates (95% CI: 0.154–0.343; P <0.001). The risk of HHcy of E2 >26.80 pg/ml group was 70.1% lower than that of E2 ≤4.99 pg/ml group after adjusting for covariates (95% CI: 0.188–0.478; P <0.001) (**Table 3**).

**Table 3:** The relationship between renal function indexes and estradiol and hyperhomocysteinemia.

Variables	Model 1 OR (95% CI) *P*	Model 2 OR (95% CI) *P*
BUN (mmol/L)	0.995 (0.993, 0.997) <0.001	0.966 (0.994, 0.997) <0.001
1.70–8.30	1	1
>8.30	0.958 (0.696, 1.319) 0.793	1.085 (0.776, 1.516) 0.634
P for trend	–	–
Scr (μmol/L)	1.028 (1.021, 1.035) <0.001	1.026 (1.020, 1.033) <0.001
<40	1	1
40–100	2.250 (0.775, 6.531) 0.136	1.710 (0.567, 5.160) 0.341
>100	23.897 (7.322, 77.990) <0.001	21.781 (6.333, 74.914) <0.001
P for trend	<0.001	<0.001
UA (μmol/L)	1.005 (1.004, 1.007) <0.001	1.005 (1.004, 1.007) <0.001
<155	1	1
155–357	3.789 (2.165, 6.633) <0.001	3.320 (1.878, 5.869) <0.001
>357	9.993 (5.586, 17.876) <0.001	8.602 (4.760, 15.547) <0.001
P for trend	<0.001	<0.001
GFR (ml/min)	0.955 (0.948, 0.961) <0.001	0.957 (0.951, 0.964) <0.001
<90	1	1
90–110	0.156 (0.110, 0.220) <0.001	0.167 (0.118, 0.238) <0.001
>110	0.131 (0.079, 0.218) <0.001	0.204 (0.119, 0.350) <0.001
P for trend	<0.001	<0.001
E_2_ (pg/ml)	0.965 (0.955, 0.975) <0.001	0.976 (0.965, 0.987) <0.001
Q1 (≤4.99)	1	1
Q2 (4.99–14.95)	0.527 (0.371, 0.749) <0.001	0.534 (0.374, 0.762) 0.001
Q3 (14.95–26.80)	0.217 (0.146, 0.321) <0.001	0.230 (0.154, 0.343) <0.001
Q4 (>26.80)	0.176 (0.116, 0.267) <0.001	0.299 (0.188, 0.478) <0.001
P for trend	<0.001	<0.001

Model 1: we did not adjust confounding factors.

Model 2: we adjusted BMI, drinking history, smoking history, living area, menopause, education.

### Mediation Analysis

**Figure 2:**
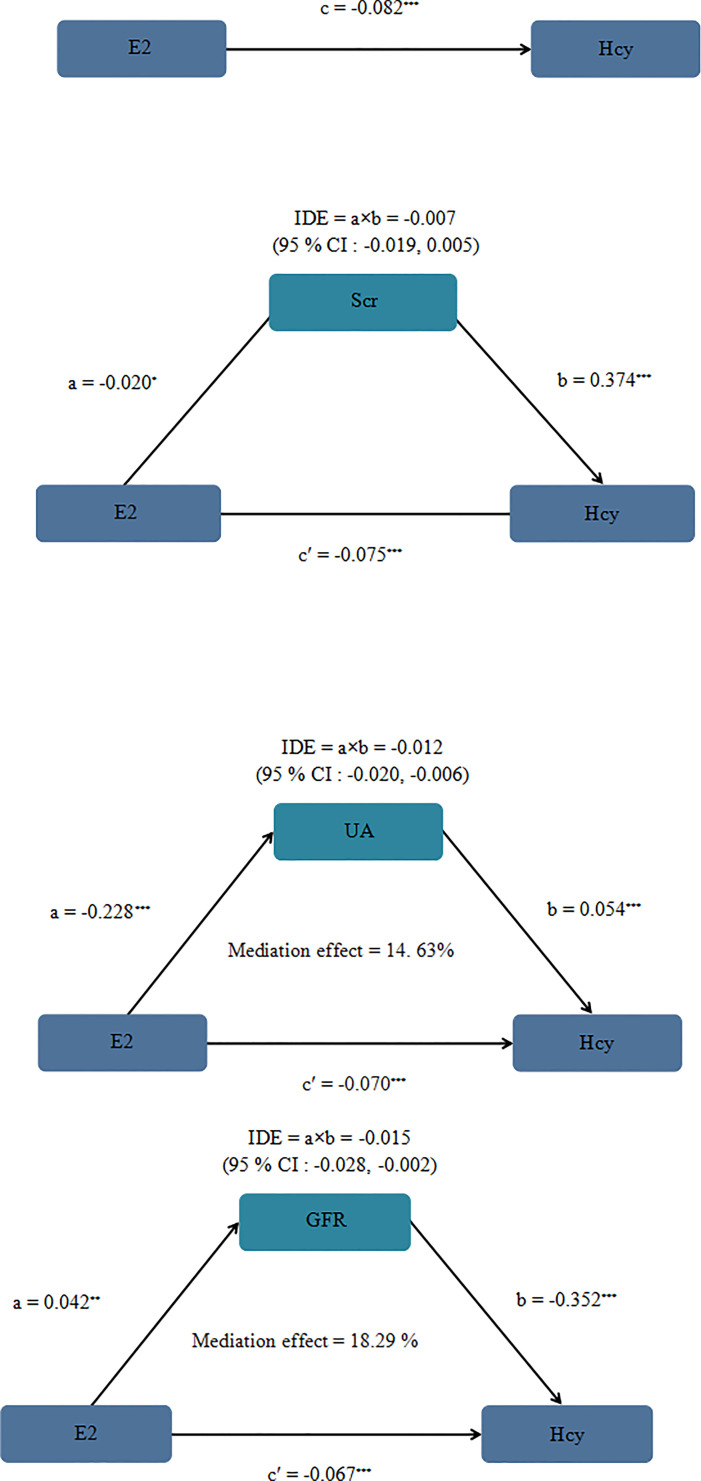
Mediation analysis model of female Scr, UA and GFR on the E2 - Hcy association after adjusting for covariates. Note: a, b, c and c' are standardized regression coefficients; a, indirect effect l; b, indirect effect 2; c, total effect; c', direct effect, IDE total indirect effect, If the 95% CI of the indirect effect includes 0, there is no mediating effect. We adjusted BMI, drinking history, smoking history, living area, menopause, education. At the same time, in the mediating effect model, we transformed Scr, UA, GFR, E2 and Hcy into natural logarithm, *P = 0.218 **P = 0.017 ***< 0.001.

We conducted the mediation analysis to explore the role of Scr, UA and GFR in the association between E2 and Hcy. **Figure 2** showed the mediation effects of Scr, UA, GFR on the E2–Hcy relationship after adjusting for covariates. The total effect of E2 on Hcy: c = −0.082 (P <0.001), the effect of E2 on Scr: a = −0.020 (P = 0.218), the effect of Scr on Hcy: b = 0.374 (P <0.001), the direct effect of E2 on Hcy: c’ = −0.075 (P <0.001). The 95% CI for the indirect effect of E2 on Hcy through the intermediate variable Scr was (−0.019, 0.005). Scr did not play a mediating role in the relationship between E2–Hcy relationship. The effect of E2 on UA: a = −0.228 (P <0.001), the effect of UA on Hcy: b = 0.054 (P <0.001), the direct effect of E2 on Hcy: c’ = −0.070 (P <0.001). The 95% CI for the indirect effect of E2 on Hcy through the intermediate variable UA was (−0.020, −0.006). The effect of E2 on GFR: a = 0.042 (P = 0.017), the effect of GFR on Hcy: b = −0.352 (P <0.001), the direct effect of E2 on Hcy: c’ = −0.067 (P <0.001). The 95% CI for the indirect effect of E2 on Hcy through the intermediate variable GFR was (−0.028, −0.002).

## Discussion

Our study found that the indirect effects of E2 on Hcy through UA and GFR accounted for 14.63 and 18.29%, respectively. Furthermore, in the mediating effect models, we can observe that UA was negatively correlated with E2, while GFR was positively correlated with E2, UA was positively correlated with Hcy, while GFR was negatively correlated with Hcy. This study suggested that menopausal female patients with abnormal UA and GFR may be more at risk of HHcy. We should pay more attention to Hcy in postmenopausal women. At the same time, abnormal kidney function will remind the body of abnormal Hcy metabolism. Regular physical examinations for postmenopausal women are helpful for early detection, early diagnosis and treatment of HHcy.

Hcy in the human body is catabolized in the liver and kidney. The metabolic process of Hcy requires folic acid and vitamins, if the levels of folic acid and vitamins in the body are insufficient, the concentration of Hcy in the body will increase and cause HHcy (17, 18). HHcy is considered a risk factor for cardiovascular and age-related diseases (19). In developed countries, elevated plasma Hcy levels are relatively rare. However, the prevalence of HHcy in the Chinese population is relatively high. Therefore, we should strengthen our attention to HHcy. A study in 2004 showed that the concentration of male Hcy was (11.4 ± 6.1) µmol/L, while the concentration of female Hcy was (9.3 ± 4.5) µmol/L. Obviously, the concentration of male Hcy was higher than that female Hcy (20). The study of Mccully also showed that the average level of female Hcy was often lower than that of male Hcy, while pre-menopausal women were lower than those of postmenopausal women (21). The above studies have shown that estrogen was closely related to Hcy. Our study showed that estradiol has a negative relationship with Hcy. At present, the mechanisms of estrogen causing Hcy changes are roughly as follows: (1) Estrogen promotes Hcy metabolism by enhancing betaine-homocysteine methyltransferase activity (13); (2) Elevated plasma Hcy may be closely related to the defects of the key enzymes CBS, CSE, methylenetetrahydrofolate reductase (MTHFR) involved in the metabolism of Hcy, and may also be closely related to the gene mutations of these key metabolic enzymes, a study showed that normal pregnant women have relatively low plasma Hcy levels during pregnancy, while HHcy is found in women with pregnancy-induced hypertension, then researchers further studied and found that the expression of CSE in the placenta was lower in women with pregnancy-induced hypertension than in normal pregnant women (22–25); (3): Studies have found that estrogen increases the binding of Hcy to low-density lipoprotein (LDL), and estrogen can also induce the production of LDL receptors in the liver, thereby promoting the binding of Hcy to LDL receptors, thereby increasing Hcy clearance (26). In the field of basic medicine, there have been many reports on the mechanism by which estrogen affects Hcy. Simultaneously, data from animal models indicated that sex hormones can mediate acute kidney injury, and estrogen was a protective factor (27, 28). Many previous clinical studies have demonstrated that Hcy was closely related to renal function indicators. Our previous cross-sectional study conducted in 24 rural and urban communities in Hunan Province also found that Hcy was related to Scr and UA. The average levels of Scr and UA in the HHcy group was significantly higher than that of the non-HHcy group (29). Data collected from patients undergoing physical examination at the First Affiliated Hospital of Guangxi Medical University in China showed that Hcy was positively correlated with UA (r = 0.393, P <0.001) (30). Data from the Hypertension Community Management Project showed that the estimated glomerular filtration rate (eGFR) of the group with Hcy ≥10 umol/L in the general population was lower than the eGFR with Hcy <10 umol/L, and female eGFR was closely related to HHcy (OR = 0.954; 0.937–0.971; P <0.001) (31). In summary, E2, Hcy and kidney function indexes are closely related. But, it is unclear whether renal function indicators play a role in the relationship between E2 and Hcy. Therefore, we carried out this case–control study to explore the inner connection of the three.

The innovation of this research lies in the following aspects: Firstly, our study adjusted some covariates in order to discover the true relationship between E2, renal indicators and HHcy. Most importantly, this study used the mediation effect model to discover the potential indirect pathways through which E2 affects Hcy. However, this study also has some limitations. This study used a case–control study and could not establish a causal relationship between E2 and HHcy. Furthermore, there may be potentially unknown confounding factors affecting the relationship between E2 and HHcy. We recruited all participants from female patients in a hospital in Hunan Province, and thus our results extrapolated to the other populations should be cautious.

### Conclusions

In this case–control study, we observed that UA and Scr were the positive relationship with Hcy, while E2 and GFR were the negative relationship with Hcy. The mediating effect models showed that UA and GFR may play the mediating role in the relationship between E2 and Hcy. E2 may indirectly affect HHcy through UA and GFR.

## Data Availability Statement

The raw data supporting the conclusions of this article will be made available by the authors, without undue reservation.

## Ethics Statement

The studies involving human participants were reviewed and approved by the Institutional Ethics Review Board of Hunan Provincial People’s Hospital. The patients/participants provided their written informed consent to participate in this study.

## Author Contributions

All authors listed have made a substantial, direct, and intellectual contribution to the work and approved it for publication.

## Funding

This study was sponsored by grants from the National Natural Science Foundation of China (Nos. 81202281, 81773530).

## Conflict of Interest

The authors declare that the research was conducted in the absence of any commercial or financial relationships that could be construed as a potential conflict of interest.

## Publisher’s Note

All claims expressed in this article are solely those of the authors and do not necessarily represent those of their affiliated organizations, or those of the publisher, the editors and the reviewers. Any product that may be evaluated in this article, or claim that may be made by its manufacturer, is not guaranteed or endorsed by the publisher.
